# Supplementary material for: Effect of Carotenoids from Phaeodactylum tricornutum on Palmitate-Treated HepG2 Cells
Source: Molecules. 2020 Jun 19;25(12):2845. doi: 10.3390/molecules25122845 (PMC7356161; doi:10.3390/molecules25122845)
Supplement: Supplementary file 1 [file molecules-25-02845-s001.pdf]

Supplementary materials

Table S1. Composition of carotenoid extract from *P. tricornutum* biomass

Components	mg/mL
**Carotenoids**	
Fucoxanthin	$2.70\ 10^{-1}$
Isomers of fucoxanthin	$2.60\ 10^{-1}$
Violaxanthin	$8.50\ 10^{-2}$
Diadinoxanthin	$8.00\ 10^{-2}$
Antheraxanthin	$1.10\ 10^{-1}$
Dinoxanthin	$1.20\ 10^{-2}$
Diatoxanthin	$7.10\ 10^{-2}$
β-carotene	$5.00\ 10^{-2}$
Others	$6.20\ 10^{-2}$
**Chlorophylls**	
Chlorophyll a	$7.00\ 10^{-3}$
Pheophytin	$2.40\ 10^{-2}$
Total pigments (mg/mL)	1.031

Table S2. Composition of total lipophilic extract from *P. tricornutum* biomass

Components	mg/mL
Lipids	13.94
**Carotenoids**	
Fucoxanthin	$2.58\ 10^{-1}$
4 keto-19'-hexanol-oxy-fucoxanthin	$5.80\ 10^{-2}$
Diadinoxanthin	$2.20\ 10^{-2}$
Other xanthophylls	$4.70\ 10^{-4}$
β-carotene	$1.16\ 10^{-2}$
**Chlorophylls**	
Chlorophyllid a	$6.70\ 10^{-1}$
Pheophytin	$9.20\ 10^{-2}$
Chlorophylls a + c	$1.46\ 10^{-1}$
Total extract	15.20

Table S3. Sequences of primers used for qRT-PCR

Gene	Sequences (5′-3′)
ACACA	F-TCGCTTTGGGGGAAATAAATG<br>R-ACCACCTACGGATAGACCGC
SOAT1	F-GAAACCGCTGTCAAAGTCC<br>R-AGGAGATGAAGAAAATCCC
ACTB	F-TGCTATCCAGGCTGTGCTATCC<br>R-GCCAGGTCCAGACGCAGG
CPT1A	F-TGGATCTGCTGTATATCCTTC<br>R-AATTGGTTTGATTCCTCCC
CPT2	F-AACCAACATGACTGTTTCTG<br>R-ATAGTGTCACTTTTTGCAGG
DGAT1	F-ATCTTCTTCTACTGGCTCTTC<br>R-AGAAGTAGGTGACAGACTC
FASN	F-ACAGGGACAACCTGGAGTTCT<br>R-CTGTGGTCCCACTTGATGAGT
LXR/NR1H3	F-GCTCCCACCGCTGCTCTC<br>R-TGCCCTTCTCAGTCTGTTCCAC
SCD	F-CAGAGGAGGTACTACAAACC<br>R-ATAAGGACGATATCCGAAGAG
SREBF1	F-AATCTGGGTTTTGTCTCTTC<br>R-AAAAGTTGTGTACCTTGTGG
18S	F-GATGCGGCGGCGTTATTCC<br>R-CTCCTGGTGGTGCCCTTCC

ACACA: acetyl-CoA carboxylase alpha, ACTB: actine beta, CPT: carnitine palmitoyltransferase, DGAT: diacylglycerol acyltransferase, FASN: fatty acid synthase, LXR: liver X receptor, NR1H3: nuclear receptor subfamily 1 group H member 3, SCD: stearoyl-CoA desaturase, SOAT: sterol O-acyltransferase, SREBF: sterol regulatory element-binding protein factor.
